# Plant medicine usage of people living with type 2 diabetes mellitus in Belize: A qualitative exploratory study

**DOI:** 10.1371/journal.pone.0289212

**Published:** 2023-08-03

**Authors:** Lindsay P. Allen, Lucia Ellis, Christophe Engleton, Valerie Lynette Valerio, Andrew R. Hatala

**Affiliations:** 1 Department of Community Health Sciences, University of Manitoba, Winnipeg, Canada; 2 Belize Diabetes Association, Belize City, Belize; Walden University, UNITED STATES

## Abstract

**Background:**

Type 2 Diabetes Mellitus (T2DM) is a primary cause of death in Belize, a low-income country with the highest rates in Central and South America. As many people in Belize cannot consistently access biomedical treatment, a reality that was exacerbated by the COVID-19 pandemic, plant medicine usage is estimated to have increased in recent years. This exploratory study seeks to understand which plants are being used, patterns of usage, and the state of patient-provider communication around this phenomenon.

**Methods:**

Implementing a Constructivist Grounded Theory qualitative design, the research team conducted 35 semi-structured interviews with adults living with T2DM, 25 informant discussions, and participant observation with field notes between February 2020 and September 2021. Data analysis followed systematized thematic coding procedures using Dedoose analytic software and iterative verification processes.

**Results:**

The findings revealed that 85.7% of participants used plants in their T2DM self-management. There were three main usage patterns, namely, exclusive plant use (31.4%), complementary plant use (42.9%), and minimal plant use (11.4%), related to factors impacting pharmaceutical usage. Almost none of participants discussed their plant medicine usage with their health care providers.

**Conclusions:**

Plant species are outlined, as are patients’ reasons for not disclosing usage to providers. There are implications for the advancement of understanding ethnobotanical medicine use for T2DM self-management and treatment in Belize and beyond.

## 1. Introduction

While the World Health Organization has warned that Type 2 Diabetes Mellitus (T2DM) is of growing concern across the globe, Belize has the highest prevalence in all Central and South America [1]. According to the 2019 World Health Ranking, Belize ranks fifth highest in prevalence of all countries in the world [1]. More than one out of six adult Belizeans live with the condition, up from one out of ten in 2010 [1]. The primary cause of death in Belize is T2DM (alongside coronary disease), causing early mortality and increased morbidity [1]. Cardiac arrests, cerebral vascular accidents, renal failures, lower limb amputations, vision damage, and vision loss are all complications associated with T2DM, with increasing rates of hospitalization in Belize, which acts to burden and strain this low-income country [2, 3].

The health system in Belize is in a transitional fledgling stage of development. The National Health Insurance, which began its roll-out as a public system in 2003, covers less than 75% of the population for primary care, leaving approximately 105,000 people to fall through the cracks [2, 4]. Private clinics and hospitals exist in Belize − for those who can afford them. This tiered health care system perpetuates an inequitable cycle as 43% percent of Belizeans live below the national poverty line (i.e., $1000 USD income per year), with 16% of these people facing extreme levels of poverty, according to a poverty assessment by to the Government of Belize [5]. Those living in poverty are both more likely to face chronicity burdens and less likely to have the resources to self-manage T2DM [6, 7]. A situational analysis for the national gender policy found that women experience higher rates of poverty than men in Belize; T2DM prevalence in women is more than twice as high than the prevalence in men [8]. The Statistical Institute of Belize describes the country as home to peoples of diverse ethnicities and cultures, with a population that is 53% Mestizo (Indigenous-Spanish), 26% Creole (British-African), 11% Mayan (Indigenous), 6% Garifuna (Indigenous-African), 3% East Indian, as well as Mennonite, Middle Eastern, Asian, and American [9, 10]. Indigenous groups in Belize − including Mayan (i.e., three distinct groups: Yucatec, Mopan, and Q’eqchi’), Garifuna, and Mestizo − account for 65% of the total national population and have high rates of T2DM [2, 9].

In the tropical forests of Central and South America, there are hundreds of plants used for treating T2DM, as well as related symptoms and complications [11, 12]. Indigenous Mayan healers, for example, utilize disease-specific and symptom-specific plant medicines, prayers, and rituals [11, 13]. Garifuna healers conduct ceremonies with ancestral prayers calling on the help of the ancestral spirits who may guide them to use specific medicinal plants, as well as to embrace behavioral and lifestyle changes that can support diabetes care and management [14, 15]. These plant medicines can often prevent and delay the “progression of diabetic related AGE formation which contribute to the development of retinopathy, cataracts, atherosclerosis, neuropathy, nephropathy, diabetic embryopathy and impaired wound healing” [11, p.505].

International efforts such as the United Nations Declaration on the Rights of Indigenous Peoples, the United Nations Convention on Biological Diversity, and the World Health Organization Traditional Medicine Strategy 2014–2023 affirm Belizeans’ rights to their traditional health practices, protections of plant medicines, and rights to health and social services without discrimination [16–18]. This is similarly affirmed by the Belize National Cultural Policy 2016–2026 that emphasizes the value of plant medicine knowledge as part of the country’s rich *Intangible Cultural Heritage* (ICH) [19]. Such policy documents call for deeper understanding of ethnomedicines and their role in health and health care provision [17–19].

A 2016 systematic review of medicinal plants used for diabetes focused on 20 studies of 40 plants which they found to be accessible, inexpensive, effective in T2DM treatment, and without adverse effects [20]. A 2018 review outlined the major properties of 14 herbal extracts as having beneficial effects in both type 1 and type 2 diabetes [21]. There have been promising results in T2DM studies specific to ginger, cinnamon, and moringa, though there are many under-researched plant medicines with significant potential [22, 23].

The purpose of this exploratory study is to describe current practices regarding: 1) the plants people in Belize are using as medicines to manage T2DM; 2) the patterns of plant medicines usage; and 3) the state of patient-provider communication around plant medicine usage. This research builds knowledge on the experiences and needs of people living with T2DM as a step toward grappling with the rising prevalence of T2DM and its life-threatening complications. It informs a policy direction toward culturally safe care in Belize and similar settings, while also providing a rationale for increased dialogue between otherwise siloed (e.g., Western, Indigenous, formal, informal) systems and practice of medicine and health care.

## 2. Materials and methods

### 2.1. Inclusivity in global research

This study was spearheaded by the Belize Diabetes Association (BDA), a non-profit organization that provides subsidized glucometers and other supports to Belizeans living with diabetes. The World Diabetes Foundation and the University of Manitoba (Canada) funded the research. Local health system designers, analysts, and administrators were collaborative and consultive partners, including employees from the Belize Ministry of Health (MoH) and the National Health Insurance (NHI) offices. The local chapter of the Pan American Health Organization (PAHO) collaborated in the development of the project. The Steering Committee included 14 people from the BDA (3), the MoH (3), PAHO (1), local health care providers (5), and administrators (2). A Belizean research coordinator, two researchers from the University of Manitoba, and three local interviewers − one of whom also specialized in data management − formed the research team. Research relationships between Belizean and Canadian team members have been ongoing since 2016.

Local Indigenous and non-Indigenous people leading the University of Belize, the National Institute for Culture and History, BDA offices, and several satellite health care services consulted in the project. Capacity-building was built into the project to help enhance skills for local and Indigenous interviewers who played an important role in data gathering. Three of the authors are local collaborators who reside in the country where the research was conducted, and they are members of the affected communities. The local Steering Committee designed the aims, questions, and priorities of the research through numerous meetings arranged by the local research coordinator. The methodology was arrived at through discussions in this series of consultations under the leadership of the local Steering Committee.

### 2.2. Ethics

All research was approved institutionally by the University of Manitoba Human Research Ethics Board (HS23313 (H2019:406)) and (HS23931 (H2020:229)). Further, the oversight of the Belizean Steering Committee required and ensured that local ethical and cultural protocols appropriate to the setting and context led the process, serving as an interim ethics committee while the country is in the process of developing − but does not yet have − a formal organizational body for these purposes. The informed consent process included an initial conversation between local liaisons and potential participants who were given research project and recruitment information. After they indicated they were interested, on a different day or later the same day, the participants presented at a designated community-use office space. Prior to commencing the interview, the interviewer and interviewee had an informed choice discussion, covering everything on the consent form verbally, with the interviewer using active listening skills and checking it made sense to the participant, with time and space allotted for questions. All participants subsequently signed written consent forms prior to interviews. All participant information was de-identified to ensure privacy and confidentiality. Additional information regarding the ethical, cultural, and scientific considerations specific to inclusivity in global research is included in the S1 Checklist.

### 2.3. Design

To address the three study objectives, we used a qualitative Constructivist Grounded Theory methodology [24, 25]. Grounded Theory methodology was deemed appropriate because it develops theory from within the parameters of the local context, iteratively comparing themes that emerge from thick data, allowing for inter-participant nuance and intra-participant complexity while evolving a clear understanding of main phenomena at play in this environment [24, 26, 27]. Qualitative semi-structured interviews were conducted with individual participants, and meetings were conducted with key informants during site visits.

### 2.4. Participants

Eligibility criteria required that study participants be adults of 18 years or older; diagnosed with T2DM; living in Belize; willing to participate; and able to converse in English (the national language), or through Mayan or Spanish interpreters (provided). Eligibility criteria for the key informants required that they be employed in health provision, health education, health administration, intangible cultural heritage, ethnobotany, plant medicine practice, or Indigenous or other cultural organizations.

### 2.5. Data collection

Snowball and purposive sampling were used to recruit 35 participants through the mobilization of the Steering Committee’s networks, to the point of data saturation and beyond [28]. Another 25 informants engaged in informal conversations at site visits throughout Belize, as per Kovach (2021) [29]. Recruitment for informants occurred through the research coordinator’s outreach via a nation-wide campaign of phone calls, emails, and in-person visits to relevant organizations. Site visits included meetings at the offices of the National Institute of Cultural Heritage, the Belize Ministry of Health, the National Health Insurance office, the Belize Diabetes Association (Punta Gorda, Dangriga, Belize City locations), the Punta Gorda Polyclinic, the San Antonio Clinic, health administration offices (Punta Gorda, Dangriga, Independence, Belmopan), the University of Belize, and in community. These meetings were not audio-recorded; for these encounters, methods included participant observation with field notes [30].

As this study was part of a larger program of research, the interview questions originally were based on the Diabetes Quality of Life Questionnaire, which was pretested for cultural saliency then modified to include additional questions about plant medicine use, spiritual and religious practices, as well as experiences of COVID-19, as per participant feedback on the measurement tool. One of the benefits of semi-structured interviews is that the participants can lead the data generation in new and surprising directions to provide insights not anticipated by the researchers–this is a sign of quality in qualitative knowledge production [24, 30, 31]. The whole question area around plant medicines was driven and developed by participants’ responses and feedback; further, Grounded Theory explicitly sets out to construct theory directly from participants lived experiences. Question areas covered in the interviews included social locations and influences, experiences and perceptions, root causes, priorities and routines, challenges, spiritual and mental aspects, formal and informal care and services, programming and education, vision of the future, and space to discuss anything else as desired. Audio-recorded, semi-structured interviews took place between February 2020 and September 2021, each lasting between 30–90 minutes. These were transcribed verbatim, incorporating memo-writing to connect data from field notes [24, 32].

### 2.6. Data analysis

Literal codes, focused codes, and analytic categories were developed by LPA, LE, and ARH in a systematized order using Dedoose analytic software [24, 32]. Literal codes refer to coding direct quotes of what is said in its immediate literal sense. Focused codes refer to denying distractions from the topic–in this case–anything not specific to plants use. Analytic categories refer to the emerging themes and subthemes. In accordance with Charmaz (2014) Constructivist Grounded Theory framework, this involved examining each line, each section, each interview, and all data from all sources together to understand the main themes and subthemes, as well as underlying meanings, patterns, processes, and assumptions, while keeping the human story central to the analysis. We reached a consensus on the coding tree through research team discussions. The rigor of the analysis was ensured through holding emic-etic discussions with the Steering Committee, practicing continuous reflexivity, questioning the emerging theory, and seeking divergent data [24, 29, 32].

## 3. Results

The interview participants and informants provided interesting data regarding their T2DM health practices. The two main themes that emerged were the widespread pervasiveness of plant medicine usage, as well as the strong tendency of non-disclosure regarding said usage in patient-provider communication.

Participants were from the five of the six districts of Belize − all but the least populated district of Orange Walk due to limitations to travel imposed during the COVID-19 pandemic. The mean average age of interviewees was 54 years old, and the sample’s age range was between 34 and 89 years old. Demographic characteristics of the sample are presented in Table 1. Of the 35 interviewees, 30 (85.7%) reported using plant medicines. There were three main usage patterns, namely, exclusive plant use (31.4% of total sample), complementary plant use (42.9% of total sample), and minimal plant use (11.4% of total sample). All but one of those participants avoided disclosing usage to their health care providers.

**Table 1:** Demographic characteristics of sample of 35 interviewees.

	Number in sample	Percentage of sample	Percentage of total population^1^	Number using plant medicines	Percentage using plant medicines
Ethnicity					
Creole	13	37%	26%	10	77%
Garifuna	6	17%	6%	6	100%
Mestizo	6	17%	53%	5	83%
East Indian	5	14%	3%	4	80%
Mayan	4	11%	11%	4	100%
American	1	3%	1%	1	100%
Sex					
Female	27	77%	50.3%	23	85%
Male	8	23%	49.7%	7	88%
Age					
50 ≥ years	10	29%	88%	9	90%
50 < years	25	71%	12%	19	76%
Occupation					
Self-employed	6	17%	41% (combined self-/employed)	5	83%
Employed	11	31%		11	100%
Unpaid domestic labour	11	31%	-	10	91%
Retired	7	20%	-	4	57%

^¹^Estimates based on 2021 population data from the Statistical Institute of Belize [10].

### 3.1. “There are so many that grow here: Pervasive usage of local plant medicines

Participants were asked to list the plants they used for their diabetes self-management. The plants that were reported are included in Table 2. Numerous plants were understood to help with directly lowering levels of blood glucose and/or alleviating T2DM symptoms, such as numbness in the extremities, sluggish circulation, skin irritations, sleep disturbances, and fatigue. Plant medicine usage was organized into three categories, namely, those who exclusively used plants, those who complemented plants with occasional pharmaceuticals, and those who used pharmaceutics primarily but complemented them with plants. Just as there was a spectrum of degrees of plant medicine usage, there existed a spectrum of practitioners, from backyard garden hobbyists to certified herbal doctors who held esteemed lectureship positions for academic audiences.

**Table 2:** Plants found in Belize that are used for type 2 diabetes self-management.

Common name	Scientific name	Part used
Aloe vera	*Aloe socotrina*	leaf
Aloe vera	*Aloe barbadensis*	leaf
Bitterbark	*Alstonia constricta*	bark
Cinnamon	*Cinnamomum zeylanicum*	bark
Coconut	*Cocos nucifera*	nut, meat, milk
Fever grass	*Cymbopogon densiflorus*	leaf
Ginger	*Zingiber officinale*	root
Gumbolimbo	*Bursera simaruba*	bark, leaf
Jackass bitters	*Neurolaena lobata*	leaf, root
Lime	*Citrus aurantifolia*	leaf, seed
Mango	*Mangifera indica*	bark, leaf
Moringa	*Moringa oleifera*	leaf
Neem	*Azadirachta indica*	bark, leaf
Noni	*Morinda citrifolia*	leaf, root
Oil nut	*Ricinus communis*	leaf, seed
Okra	*Abelmoschus esculentus*	leaf, seed
Papaya	*Carica papaya*	fruit, leaf, seed, root
Sage	*Salvia officinalis*	leaf
Sour sop	*Annona muricata*	fruit, leaf
Tumeric	*Curcuma longa*	root
Vervain	*Verbana hastat*	leaf
Vervain	*Stachytarpheta jamaicensis*	leaf

#### 3.1.1. Exclusive plant use

The first pattern of usage that emerged from the data analysis was the category of 11 participants who exclusively used plants for T2DM. Creole Woman 3, for example, stated that she refused to use pharmaceuticals. With the help of a practitioner who was both a medical doctor and a certified herbalist, Creole Woman 3 sought to control her blood glucose levels with herbal preparations:

“[I use] some herbs that… for the past two years I’ve been drinking three times a day. She [the herbal doctor] makes it. Every six months or so, I do a complete blood test, and she checks the A1C. It has gone down. One time, it used to be like two hundred. Whoo! For ages… I don’t take any pills. No. I don’t like pills. I don’t even take Tylenol.”

Creole Woman 3 could not specify which combination of plants she was using, having relied on her herbal doctor − who was a retired medical doctor–to know. Despite not knowing the details, she was certain that they helped her manage her T2DM symptoms.

Garifuna Man 2 explained that he had tried various T2DM pharmaceuticals but had suffered from a long process of incorrect discernment of suitable dosages, and he grew wary of the expense and the side effects, as he found the pharmaceutical pathway had worsened his quality of life. He stopped using pharmaceuticals altogether and made his own eyedrops and pain medications from local plants. He shared: “That start to make my eyes heal… All the medication I was getting, it wasn’t working… I was using, like three medications… for the pain… Herbs, for that purpose, they are very, very effective.”

Participants who fell in this category still interacted with their physicians, but they assumed more autonomy over their T2DM management, using plant medicines they found accessible. Economic barriers, mistrust of pharmaceuticals, dislike of side effects, and perceived lack of medication efficacy (perhaps compounded by irregular access) were all factors.

#### 3.1.2. Complementary plant use

The second category included people who preferred plants but complemented them with pharmaceuticals in 15 participants, such as Garifuna Woman 2. She reported that she used pharmaceuticals when they were available in her local clinic, but supply was inconsistent, so she accumulated plant medicines from the garden and trees in her yard, as well as from her neighborhood and social networks. The plants (raw matter or prepared) cost her less money and were more reliably accessible than pharmaceuticals. Garifuna Woman 2 pointed to nearby, imparting:

“I have fever grass tea that I buy, and sage… I use moringa… It’s a flower, white. That’s it right there. And the soursop too…Whenever somebody else come tell me about the herbs, I’ll buy it, and I drink it, and it helps.”

She was not concerned with the lack of regulatory bodies or standardized dosages; she placed her faith in plant medicines categorically, trusting in informal knowledge-sharing, and not necessarily distinguishing between different plants. Trust in the source of plant medicines (e.g., as ‘of-the-land,’ ‘natural,’ ‘God-given’) played a significant role in participants’ relationships between self-and-medicine. When they could interact directly with plants (e.g., locating, gathering, preparing), they felt more empowered than when they tried to access pharmaceuticals only to be confronted by multiple barriers (e.g., supply-chain, economic).

Garifuna Man 1 had been diagnosed only days before his interview. He stated that he wanted to use insulin for as short a time as possible until he adjusted; he hoped this would only take a matter of weeks. He shared: “I am on insulin, but I’m trying to ween myself off of that. I’ll be trying neem.” He had already contacted a well-known Belizean herbal doctor of his own cultural heritage (Garifuna) to control his blood glucose because he considered plants to be safe, accessible, and effective. He explained that he did not want to depend on pharmaceutical companies from foreign countries but rather on medicines found on the land around him.

Creole woman 5 described her preferences for herbal medicines that empowered her by saving her money and enabling her to avoid clinics, to which she expressed a strong aversion. She lived near numerous plants:

“I take a lot of natural stuff. I use turmeric, ginger, cinnamon, moringa, things like that… I’m using it as a powder, but my neighbor has a lot of moringa trees, so, I will go and pick the seeds because the seeds are good too. And the leaf. I would dry it, as you dry tea. Then you drink that… Well, I haven’t been to the doctor for diabetes in a long time.”

Informants shared how during the COVID-19 pandemic, clinics drastically reduced their hours and services. Most participants reported not seeing a health professional for as much as two years, whereas routine T2DM care is typically set to the standard of a check-up once per three months including a A1C blood glucose–supplies for which were strained even before the pandemic. The void in care prompted deepening reliance on local plant medicines.

#### 3.1.3. Minimal plant use

The third category of usage consisted of 4 participants who were dedicated to taking prescription medications but still used plant medicines on occasion. There was little known about contraindications or interactions with her medications or otherwise. Garifuna Woman 3, for example, had a complex case of T2DM with co-morbidities. She relayed that she depended on her prescriptions but also used plant medicines for blood glucose levels, dengue fever, and high blood pressure without discerning the effects of mixing medicines. Garifuna Woman 2, a retired nurse with a relatively new diagnosis, said that she was committed to following her doctor’s orders, including taking daily medications. She also kept an herb in her garden and regularly used plant medicines growing in the surrounding environment. She described:

“You hear about all types of herbal medication that you can take along with your pills. All kind of herbs… I drink the noni… for diabetes…the moringa, …the gumbolimbo bark to make tea with it. This tree they tell me about it, and I use it. But I don’t know if it helps because I still take my medication… I am careful. My thing is, if I don’t take my diabetic medication, my organs will damage quick. So, I stick to my pills… I stick to it. I take the herbal tea as a complement, but I stick to my medication.”

Across all three categories, there was near consensus on the perceived safety of using plants as T2DM medicine. One participant, East Indian Man 3, stood out. He reported that though he used herbs, he had concerns regarding the pervasive phenomena of self-prescribing.

“Everybody has problems with diabetes. A lot of people here have problems with it, and they mentioned that they are drinking herbs, …but they don’t come and check again. They say that is helping, …[but] they still have to come in [to get checked].”

He worried about unknown side effects of plant medicines, saying: “That is frightening because there is not anyone to guide you to say only drink this amount of bush medicine. You need someone to give you specifics."

Key informants agreed that plant medicine usage was pervasive. They wanted to see more support for botanical knowledge production and dissemination (e.g., to promote initiatives in backyard gardening, medicine and food security and sovereignty, sustainable eco-tourism, higher learning opportunities). The COVID-19 pandemic heightened the desire for improving domestic food and medicine systems, as border and port closures exacerbated pre-existing supply shortages (e.g., T2DM medications, glucometers, glucose test strips, quality proteins, complex carbohydrates). One key informant (Garifuna) kept a bookcase of herbal preparations ready for the many relations who came to her for informal health care–she actively resisted feelings of helplessness that would otherwise subsume her, feeling happy to be able to supply them some relief for their ailments at no cost.

There was another dimension to the belief and trust in plant medicines. East Indian Man 4 said: “The tree is the healing of the nation. I believe there is a bush for every sickness on this earth.” Similarly, Mestizo Woman 2 expressed: “God put all trees and plants on this earth because all of them have something for all kinds of sickness. I think natural remedies is the best medicine.” Plant medicines were experienced as, believed to be, and valued as empowering and life-affirming, culturally and spiritually congruent, and dependably available even under the toughest of COVID-19 restrictions.

### 3.2. “They don’t really listen:” Difficulty with patient-provider communication

The second main theme that emerged was that of difficult communication between health care providers and people living with T2DM in Belize. While most participants told interviewers that they used plant medicines, they did not disclose this pertinent health information to their care providers. When asked if they talked to their doctors about using plant medicines, participants across the three categories of usage typically did not. Mestizo Woman 3, for example, elaborated on the unspoken norm of ‘don’t ask, don’t tell:’

“I take moringa. I take it every morning for tea. I feel good when I take it. No, I don’t tell the doctor, and he did not ask me if I take it. But when I take it, I don’t have any problems.”

There were participants who shared that they believed that their provider would be unreceptive and even angry if they told them, and previous negative encounters led to patient fear of disclosure and omissions in reporting. Though many of the plant medicines have not been studied via double blind randomized controlled trials, participants were not concerned about this lack of evidence. Unbothered, they were not waiting for external validation, nor did they feel empirical evidence was crucial to their usage and understanding of efficacy.

In some instances, it was clear that communication was already strained by cultural differences and class tensions. Creole Woman 1 described her dilemma with her doctor (of a different culture and ethnicity):

“I always want to ask her, but she’s so delicate, you have to think what you [are] asking her… She doesn’t tell me anything.” She elaborated on how she felt that she was perceived as ignorant and inferior, to the point she had stopped asking her doctor questions altogether. Garifuna Man 2, told a story about getting the run-around for years when trying to access T2DM services, and who had ended up partially blind due to botched surgeries and complications, surmised: “Most of the doctors…they don’t really listen to the patient… or explain what exactly is the problem.”

Seven participants advocated for more active listening (e.g., asking for clarification, reflecting) to ensure better patient-provider communication. Garifuna Man 1 made a point of booking multiple appointments close together to ensure time to ask his questions. In instances when his physician was preoccupied during his appointment with phone calls and paperwork, he waited in the office until the end of the day to ask his questions. Informants from health offices often cited shortages of medical supplies, educational pamphlets, nutritionists and other specialists, and staff in general as part of the larger issue.

Participants wanted more open dialogue about the nuances of managing multiple prescriptions and medicinal botanicals. Garifuna Man 2 described an issue he had when he asked his provider about a new prescription. He had read about potential adverse effects online, feeling concerned when he learned the drug could weaken the immune system. Rather than engage in an informative discussion, his doctor got angry. He said: “That’s why I decided not to use it. When I explain to her, she get very mad…[so] I just told her I would use it, but I never use it.”

Mestizo Man 1 tried an anti-diabetic prescription but felt the side effects were extreme with significant weight gain and low blood pressure. He started a conversation about it with his doctor, but there was no follow-up. The physician did not inquire for details, nor explain the pharmacological effects, so he left the experience feeling confusion, frustration, and disillusionment with the medical establishment in general. This convinced him he needed to take matters into his own hands, and he began preparing home remedies from locally available plants, thus impacting the trajectory of his T2DM care.

When asked about his interactions with his doctor, East Indian Man 1 expressed deep gratitude for physicians who have worked hard for years to learn about medicine and to share what they learn with others. He went on to say: “I just would like them to keep on advising us. Especially people with diabetes. How to go on living this life.” While a couple people expressed negative experiences being scolded by doctors upon disclosure of plant usage, East Indian Woman 1 represented an exception as she found it helpful for open communication when a doctor told her it was alright to take the herbs she was taking, but that she needed to take the pills too.

## 4. Discussion

This study identified some of the plants that people are using for T2DM in Belize, their patterns of usage, and some difficulty with patient-provider communication on the subject, indicative of the larger disconnection between the biomedicine and ethnomedicine.

A 2021 study on T2DM self-management in Eastern Ethiopia pointed out that COVID-19-related medication shortages bolstered patient preference for herbal medicines, similar to our findings; however, the Ethiopia study discounted any validity to plant medicines [33]. While Letta and colleagues (2021) problematize patient preference of using herbs over pills, and they see the solution to be a sustainable–pandemic circumstances notwithstanding–supply of medications, this study suggests there is also potential in locally accessible plant medicines, especially with more (necessarily culturally safe) research and development. Plant medicines have significance to intangible cultural heritage and to inexpensive community-based care, while pharmaceutical medications are often originally derived from plants, thus discounting them categorically unwarranted [26, 34–37].

A 2019 international review found cultural safety to be a prerequisite of health equity for Indigenous people, inclusive of access to traditional plant medicines [38]. While the trend over the past thirty years of mandating cultural competency training for healthcare professionals is incrementally helpful, bolstering cultural safety is far more crucial because it goes further than learning about the patient’s culture; it addresses the underlying power imbalances that are otherwise continually perpetuated via societal institutions and within provider-patient communication [38]. Interventions that are grounded in patients’ culturally-specific understandings of health positively impact T2DM outcomes [39].

This study’s findings suggest that if cultural safety around plant medicine usage were developed in Belize, then patients could benefit in several ways: reducing fear of disclosure; improving patient-provider trust and communication; facilitating health literacy and education; and enhancing quality of care and patient satisfaction. Bringing people together from various positions on the spectrum of formal to informal health care could be beneficial in promoting dialogue, deepening understanding, enabling problem-solving, and propelling innovation, research, and development initiatives with the common goals of improving health and care for Belizeans living with T2DM.

Ethnopharmacological literature has begun to reveal that there are hundreds of medicinal plants growing in the forests, pastures, wetlands, mangroves, and other diverse ecosystems of Central and South America with applications for T2DM, its sequelae, and its symptoms [11, 12]. Plant medicines have profound cultural and spiritual significance to local populations, and there are many gaps in the literature regarding location- and culture-specific variability of application and knowledge [11, 12]. There have been studies in Trinidad and Tobago that found overlap in plant medicine usage for T2DM, namely, regarding the aloe, coco, and papaya plants [36, 40]. In a synthesis of 25 meta-analyses of plants that are used around the world and that have been undergone controlled experiments for T2DM medicinal efficacy, those with the largest effects on HbA1c blood glucose tests were aloe vera leaf gel, psyllium fiber, and fenugreek seeds [41]. Numerous plant medicines were found to reduce fasting plasma glucose tests [41]. While no serious negative effects have been found, many plants are still unstudied, in terms of efficacy or otherwise [41] Three of the plants we found to be used for T2DM in Belize, namely, aloe, cinnamon, and ginger have been studied, with aloe showing the most consistently promising results [41]. Our findings contribute to the list of T2DM-relevant plant medicines worthy of further inquiry, as well as an exploration of surrounding issues of communication, trust, and disclosure.

An HIV/AIDS case study in Belize that found that Mayans often felt torn between the traditional Indigenous healing system (inclusive of plant medicine) and biomedicine [13]. While a lot of traditional knowledge has been lost through processes of colonial suppression and the dominance of biomedicine, two systems of medicine can engage areas of mutuality or “windows of compatibility,” building on work by Dickinson, 2008 [42]. Our study reaffirmed that there are two medicine systems, that people feel torn between them, and that this tension represents an unnamed barrier to health and health care. As this tension requires patients to maintain the unspoken segregation of systems, it represents yet another burden. People must choose between a culturally safe informal system and a medically established, more resourced system, or else they must hide their participation in one from the practitioners of the other. This phenomenon extended beyond the Mayan to diverse Belizeans.

Waldram and Hatala (2015) found that while traditional Indigenous healers welcomed dialogue with biomedicine practitioners, this had never been clearly reciprocated, thus this is what is needed for bridge-building moving forward. Given that practitioners of both systems are working with overlapping patients, it follows that they would have common motivation to improve the state of the relationship and communication between systems. Case studies on intercultural health initiatives in Guatemala, Chile, Colombia, Ecuador, and Suriname defined the required shared principles of mutual respect (e.g., between individual practitioners, systems of medicine) and openness (e.g., to being in relationship, adapting to new learnings) [43]. In integrative scenarios in international settings, bridge building happens when health care providers have trained in cultural safety while traditional healers have coordinated associations to communicate their aims, needs, and standards to the formal health care system [13, 39, 44]. Cross-cultural medical collaboration could prove an important direction not only for treating T2DM, but also for co-morbidities, including mental health conditions such as anxiety, depression, substance misuse, and post-traumatic stress disorder [45, 46].

These type of innovations require resources to develop, they implicate surrounding legalities, and they necessitate thoughtful selection of practice models, role clarity, and appropriate adjoining agreements [43]. In various international contexts, Indigenous-led intercultural health services have demonstrated benefits including improved services and programs uptake, faster remote-setting urgent response, and decreased all-cause mortality [39, 44]. Similar interventions have been shown to improve multiple indicators, such as access to prenatal care, remote maternal and infant birth outcomes, childhood vaccination rates, patient trust in providers, patient satisfaction, community and cultural pride, and addiction treatment retention and drug urine tests [44]. Reduced malnutrition, fetal alcohol syndrome incidence, HIV mortality rates, emergency department use, and ER staff turnover have also been resultant of such innovations [44]. Key informants expressed enthusiasm for this direction in Belize, stating that inaccessibility of exported medications and unmet medical needs have become more pressing than ever since COVID-19, thus they merit more inquiry in and of themselves.

Belize has held in preservation many of its forests, flora, and intangible cultural heritage, and thus fosters the protection of plant medicines [11, 47]. Eco-tourism was an important industry to Belize, and though it suffered during travel restrictions during the COVID-19 pandemic, many local people were interested in revitalizing and expanding economic, educational, and medicinal opportunities in this industry. While 25% of Belize citizens still do not have access to public health care [2, 9], traditional Indigenous healers and other plant medicine practitioners are an important component of the health care context. People continue to turn to those knowledgeable in plant medicines. A large majority of the participants in this study used plant medicines in their diabetes self-management routines.

As women are more likely to experience poverty, stress, and T2DM − while also carrying a disproportionately heavier burden of unpaid caregiving work − than men in Belize [8], more research is needed to understand how gender and chronicity are interacting with plant medicine usage. A limitation of the study was the lack of intersectional analysis regarding how poverty and gender interact with plant usage and health care access. The purposive and snowball sampling did not ensure a statistically representative sample.

More research is needed to understand how specific plant medicines are being used, their efficacies and applications, how these affect T2DM outcomes, relevant guidelines for health care providers, how stakeholders can collaborate in health-promotion efforts, and best practices for community engagement in ethnoecology research in Belize. In the big picture of addressing the rising T2DM prevalence, underlying issues of poverty and inequity need to be addressed, as does access to health care for the underserved and unserved populations. Stakeholders need to implement public health campaigns that connect biomedicine and ethnomedicine to address T2DM via a culturally safe approach. This exploratory qualitative study was a preliminary step in beginning to address these knowledge gaps and provide direction for future research and policy priorities.

## 5. Conclusions

This study’s research question area was driven by people living with T2DM in Belize, via the impact of applying Grounded Theory methodology which centers to amplifies participants’ voices with aims of social justice and health equity [48]. The pervasiveness of plant medicine usage suggests many possible implications for public health and clinical practice, as well directions for ethnomedicinal diabetes research. These directions would require local leadership and community participation to prevent exploitation, appropriation, and cultural harm [46]. Recommendations include improving patient-provider communication, cultural safety in health services, and enhanced partnerships across informal and formal health systems.

## Supporting information

S1 File (DOCX)

S1 Checklist Inclusivity in global research. (DOCX)
