# Supplementary material for: Plant medicine usage of people living with type 2 diabetes mellitus in Belize: A qualitative exploratory study
Source: PLoS One. 2023 Aug 3;18(8):e0289212. doi: 10.1371/journal.pone.0289212 (PMC10399819; doi:10.1371/journal.pone.0289212)
Supplement: S1 File — (DOCX) [file pone.0289212.s001.docx]

## Ethics approval and consent to participate/ Institutional Review Board Statement:

All research was approved by the University of Manitoba Human Research Ethics Board (HS23313 (H2019:406)) and (HS23931 (H2020:229)). Further, the oversight of the Belizean Steering Committee required and ensured that local ethical and cultural protocols appropriate to the setting and context led the process, serving as an interim ethics committee while the country is in the process of developing - but does not yet have - a formal organizational body for these purposes. This study was spearheaded by the Belize Diabetes Association (BDA), a non-profit organization that provides subsidized glucometers and other supports to Belizeans living with diabetes. The World Diabetes Foundation and the University of Manitoba (Canada) funded the research. The Belize Ministry of Health (MoH) and the Pan American Health Organization (PAHO) acted in collaboration. The Steering Committee included 14 people from the BDA (3), the MoH (3), PAHO (1), local health care providers (5), and administrators (2). A Belizean research coordinator, two researchers from the University of Manitoba, and three local interviewers formed the research team. Research relationships between Belizean and Canadian team members were ongoing since 2016.

Informed Consent Statement: All participants received verbal informed choice discussions then subsequently signed written consent forms prior to interviews.
