# Supplementary material for: Plant medicine usage of people living with type 2 diabetes mellitus in Belize: A qualitative exploratory study
Source: PLoS One. 2023 Aug 3;18(8):e0289212. doi: 10.1371/journal.pone.0289212 (PMC10399819; doi:10.1371/journal.pone.0289212)
Supplement: S1 Checklist — (DOCX) [file pone.0289212.s002.docx]

Inclusivity in global research

PLOS’ policy on inclusivity in global research aims to improve transparency in the reporting of research performed outside of researchers’ own country or community and ensures that PLOS publications reporting global research adhere to high standards for research ethics and authorship. Authors of relevant research articles may be asked to complete the questionnaire below, which outlines ethical, cultural, and scientific considerations specific to inclusivity in global research. This questionnaire may be requested when researchers have travelled to a different country to conduct research, if research uses samples collected in another country, research with Indigenous populations or their lands, or if research is on cultural artefacts. Researchers travelling to another country solely to use laboratory equipment will not normally be required to complete the questionnaire. However, the questionnaire can be requested at the journal’s discretion for any submission – if you have been requested to complete this questionnaire by the PLOS journal you submitted to, please do so.

Please complete the questionnaire below and include this as a Supporting Information file with your manuscript. Note that if your paper is accepted for publication, this checklist will be published with your article in the supporting information files. Please ensure that you reference the checklist in the main body of your manuscript. We suggest adding a subsection ‘Inclusivity in global research’ to your Methods section and adding the following sentence: “Additional information regarding the ethical, cultural, and scientific considerations specific to inclusivity in global research is included in the Supporting Information (SX Checklist)”

The questions have been designed to be applicable to a wide range of study types, and there are subsections for both human subjects research and non-human subjects research. If any of the questions are not relevant to your research please mark them as “N/A” as appropriate.

**Ethical considerations, permits and authorship**

*This section is applicable to all research types.*

Provide details as to who granted permissions and/or consent for the study to take place in the Methods section of your manuscript. This should include the names of **all** ethics boards, governmental organizations, community leaders or other bodies that provided approval for the study. If individuals provided approval refer to these people by their role or title but do not list their name(s).

Reported on page number: 6-7

If there were any deviations from the study protocol after approval was obtained please provide details of these changes in the Methods section of your manuscript.
Did this study involve local collaborators that are residents of the country where the research was conducted or members of the community studied? If you do not have any authors from said communities, please provide an explanation for this below.

Reported on page number: N/A

Three of the authors are local collaborators who reside in the country where the research was conducted, and they are members of the affected communities. The study was initiated by the leaders of the Belize Diabetes Association (BDA) which is a locally run non-profit organization that supports Belizeans who live with diabetes. Local health system designers, analysts, and administrators were collaborative and consultive partners, namely, employees from the Belize Ministry of Health and the National Health Insurance offices. The local chapter of the Pan American Health Organization collaborated in the development of the project. Local Indigenous and non-Indigenous people leading the University of Belize, the National Institute for Culture and History, BDA offices, and several satellite health care services consulted in the project. Capacity-building was built into the project to help enhance skills for local and Indigenous interviewers who played an important role in data gathering. A local Steering Committee conducted oversight to ensure local ethical and cultural protocols were carried out appropriately. This Steering Committee served as an interim ethics committee while the country is in the process of developing a formal organizational body for these purposes, which it currently does not have.

Everyone listed as an author should meet PLOS’ criteria for authorship and all individuals who meet these criteria should be included in the author byline, rather than the acknowledgements. Authorship criteria is based on the International Committee of Medical Journal Editors (ICMJE) Uniform Requirements for Manuscripts Submitted to Biomedical Journals - for further information please see here: <https://journals.plos.org/plosone/s/authorship>.

This project was not focused on any one Indigenous community or cultural group, and there were no formal or informal leadership or political bodies to request permission from in that sense. This was a nation-wide project open to any Belizean who fit the study criteria (e.g., living with type 2 diabetes). It was approved by key partner and stakeholder, the locally run Belize Diabetes Association, which includes Indigenous people in its leadership and has offices in several parts of Belize also run by Indigenous and local people. Local Indigenous groups were made aware of the project, invited to collaborate and/or consult in the current or future projects, and/or to provide feedback as desired via informal contacts and formal cultural associations. The local Indigenous Research Coordinator has an excellent reputation for her community health work over decades and maintained excellent relationships with contacts throughout communities in all the regions of Belize throughout the project. The project received approvals from the Belizean Ministry of Health and the National Health Insurance employees and officiants. It received approval from local people in each local health service branch and satellite office. It received approval from individual participants in the form of recorded verbal informed consent discussions and written informed consent forms. Participants were asked to speak on behalf of themselves, not on behalf of their Peoples, nor as designated cultural representatives.

**Human subjects research (e.g. health research, medical research, cross-cultural psychology)**

Did you obtain written informed consent from a representative of the local community or region before the research took place? How did you establish who speaks for the community? Details of written informed consent obtained from study participants should be reported separately in the Methods section of your manuscript.

How did members of the local community provide input on the aims of the research investigation, its methodology, and its anticipated outcome(s)?

When engaging with the local community, how did you ensure that the informed consent documents and other materials could be understood by local stakeholders?

The local Steering Committee designed the aims, questions, and priorities of the research through numerous meetings and consultations arranged by the local research coordinator. The methodology was arrived at through discussions in this series of meetings and consultations under the leadership of the local Steering Committee.

The informed consent process included an initial conversation between local contact people and potential participants who were given research and recruitment information. After they indicated they were interested, on a different day or later the same day, the participants presented themselves to the interviewer. Prior to commencing the interview, the interviewer and interviewee had an informed choice discussion, covering everything on the consent form verbally, with the interviewer using active listening skills and checking it made sense to the participant, with time and space allotted for questions.

Will the findings of the research be made available in an understandable format to stakeholders in the community where the study was conducted (e.g. via a presentation, summary report, copies of publications, etc.)? Please provide details of how this will be achieved.

There was a presentation accessible online across Belize during COVID-19 to summarize the project methods and findings. There were follow-ups conducted by the local research coordinator. Once it is published, the article will be made available to local stakeholders via the local research coordinator.

**Non-human subjects research using specimens/ animals collected as part of the study, or those housed in archival collections. Examples include archaeology, paleontology, botany and zoology.**

Did the permission you obtained from a local authority to perform the study include an agreement on access to outputs and benefit sharing? This may include procedures to enable fair distribution of the benefits and resources arising from the research performed. Please include any details of Prior Informed Consent and Benefit Sharing Agreements obtained. These may be required by field-specific regulations, for example the Convention on Biological Diversity (CBD) and the associated Nagoya Protocol.

N/A

If the material used in your study was imported, please A) provide the year it was imported and B) indicate whether permits were obtained to import/export the materials used, C) provide details of any permits obtained. If this information is not available, please indicate this.

N/A

If you used archival specimens, please state how the material used in your study was acquired by the institute it is held in and provide details of any permits obtained for the original excavations/ sample collection. If this information is not available, please indicate this.

N/A

How was the potential cultural significance of the materials collected in your study to local communities considered in your research design? Were Indigenous peoples and/or local researchers and institutions involved with archaeological excavations / collection of specimens? If so, please provide a description of their involvement.

N/A

If your manuscript includes photographs of human remains please indicate whether authors obtained permission from descendants or affiliated cultural communities to do so.

N/A
